# Bisphenol A, Bisphenol S, and 4-Hydro​xyphenyl 4-Isopro​oxyphenyl​sulfone (BPSIP) in Urine and Blood of Cashiers

**DOI:** 10.1289/ehp.1409427

**Published:** 2015-08-25

**Authors:** Kristina A. Thayer, Kyla W. Taylor, Stavros Garantziotis, Shepherd H. Schurman, Grace E. Kissling, Dawn Hunt, Brenda Herbert, Rebecca Church, Rachael Jankowich, Mona I. Churchwell, Richard C. Scheri, Linda S. Birnbaum, John R. Bucher

**Affiliations:** 1 Division of the National Toxicology Program,; 2 Clinical Research Unit, and; 3 Biostatistics Branch, National Institute of Environmental Health Sciences, National Institutes of Health (NIH), Department of Health and Human Services (DHHS), Research Triangle Park, North Carolina, USA; 4 Division of Biochemical Toxicology, National Center for Toxicological Research, U.S. Food & Drug Administration, Jefferson, Arkansas, USA; 5 National Cancer Institute, NIH, DHHS, Research Triangle Park, North Carolina, USA

## Abstract

**Background:**

Bisphenol A (BPA) is a high-production-volume chemical associated with a wide range of health outcomes in animal and human studies. BPA is used as a developer in thermal paper products, including cash register receipt paper; however, little is known about exposure of cashiers to BPA and alternative compounds in receipt paper.

**Objective:**

We determined whether handling receipt paper results in measurable absorption of BPA or the BPA alternatives bisphenol S (BPS) and 4-hydroxyphenyl 4-isoprooxyphenylsulfone (BPSIP).

**Methods:**

Cashiers (n = 77) and non-cashiers (n = 25) were recruited from the Raleigh–Durham–Chapel Hill region of North Carolina during 2011–2013. Receipts were analyzed for the presence of BPA or alternatives considered for use in thermal paper. In cashiers, total urine and serum BPA, BPS, and BPSIP levels in post-shift samples (collected ≤ 2 hr after completing a shift) were compared with pre-shift samples. Levels of these compounds in urine from cashiers were compared to levels in urine from non-cashiers.

**Results:**

Each receipt contained 1–2% by weight of the paper of BPA, BPS, or BPSIP. The post-shift geometric mean total urinary BPS concentration was significantly higher than the pre-shift mean in 33 cashiers who handled receipts containing BPS. The mean urine BPA concentrations in 31 cashiers who handled BPA receipts were as likely to decrease as to increase after a shift, but the mean post-shift concentrations were significantly higher than those in non-cashiers. BPSIP was detected more frequently in the urine of cashiers handling BPSIP receipts than in the urine of non-cashiers. Only a few cashiers had detectable levels of total BPA or BPS in serum, whereas BPSIP tended to be detected more frequently.

**Conclusions:**

Thermal receipt paper is a potential source of occupational exposure to BPA, BPS, and BPSIP.

**Citation:**

Thayer KA, Taylor KW, Garantziotis S, Schurman SH, Kissling GE, Hunt D, Herbert B, Church R, Jankowich R, Churchwell MI, Scheri RC, Birnbaum LS, Bucher JR. 2016. Bisphenol A, bisphenol S, and 4-hydro​xyphenyl 4-isopro​oxyphenyl​sulfone (BPSIP) in urine and blood of cashiers. Environ Health Perspect 124:437–444; http://dx.doi.org/10.1289/ehp.1409427

## Introduction

**Figure 1:**
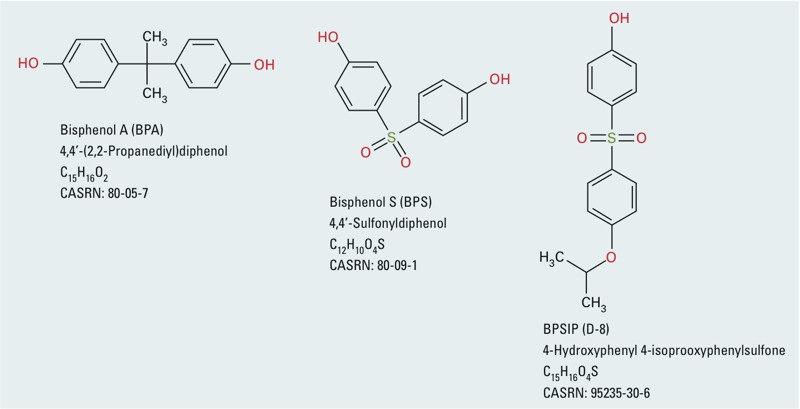
Chemical structures, common names, systematic names, molecular formulas, and CAS numbers of BPA, BPS, and BPSIP.

Human exposure to bisphenol A (BPA) is widespread [European Food Safety Authority (EFSA) 2013], and BPA is associated with a wide range of health outcomes in animal and human studies [Food and Agriculture Organization of the United Nations/World Health Organization (FAO/WHO) 2011]. Based on its use in the manufacture of polycarbonate plastic and epoxy resins in food packaging containers and can linings, the primary route of exposure to BPA in the human population is thought to be oral; however, other sources of exposure have also been identified. For example, BPA and BPA analogues such as bisphenol S (BPS) are used as dye developers in thermal paper products, including cash register receipt paper [EFSA 2013; Liao et al. 2012c; U.S. Environmental Protection Agency (EPA) 2014]. Other chemicals have been identified as potential alternatives to BPA in thermal paper in the U.S. EPA Design for Environment (DfE) report “Bisphenol A alternatives in paper”; such alternatives include the BPS derivative 4-hydroxyphenyl 4-isoprooxyphenylsulfone (also called BPSIP or “D-8”), although the extent to which they are being used is not known (U.S. EPA 2014) (Figure 1). Notably, the goal of the DfE report was not to recommend a safe alternative(s) to BPA but rather to summarize information on potential hazards. If thermal paper contributes to increased uptake of BPA or its analogues, then a study of occupationally exposed individuals such as cashiers may be informative.

Very few biomonitoring data are available to determine whether cashiers have higher urine or blood levels of BPA or BPA alternatives than non-cashiers. There are reports of elevated urinary BPA levels in cashiers participating in the Health Outcomes and Measures of the Environment Study (Braun et al. 2011) and in people who reported working in retail industries in the 2003–2004 National Health and Nutrition Examination Survey (NHANES) (Lunder et al. 2010). However, neither of these studies specifically collected samples near the time of the participants’ work shifts. Studies to simulate exposure in cashiers from dermal contact have suggested that an extensive amount of contact is needed to detect a post-handling increase in BPA (Ehrlich et al. 2014; Porras et al. 2014), at least with dry hands. Wet conditions appear to facilitate skin transfer (Biedermann et al. 2010). The simulation studies only focus on dermal exposure, but other possible routes of exposure to BPA for cashiers include hand-to-mouth ingestion after handling receipts and inhalation of dust containing the developers. Use of ethanol-based hand sanitizers has been shown to enhance the transfer of BPA from the receipt to the surface of the hand (Hormann et al. 2014).

The main objective of this study was to test the hypothesis that occupational exposure to thermal receipt paper results in increased urine and/or serum levels of BPA or its analogues in cashiers when measured shortly after they complete a work shift compared with levels measured ≥ 24 hr after completing a shift. We also analyzed samples of receipt paper to verify potential exposures and to determine whether theoretical BPA alternatives identified in a recent report from the U.S. EPA are actually in use (U.S. EPA 2014). We matched the analyses of biospecimens from cashiers with analyses of receipt paper samples provided by the cashiers. Thus, we were able to evaluate the association of levels in urine and serum with detection of BPA, BPS, or BPSIP in thermal receipt paper. We also compared levels of these compounds in urine from cashiers with those in urine from non-cashiers.

## Methods

*Participant recruitment and selection.* Cashiers (required to be > 18 years old, not pregnant, and working at a cash register for at least 20 hr/week) and non-cashiers were recruited by open advertisement from the Raleigh–Durham–Chapel Hill region of North Carolina during June 2011–September 2013. Cashiers were asked to provide proof of employment as a cashier, and all participants were asked to provide a medical history including disease status, current medications, alcohol and cigarette use, and food and drink consumption during the previous 24 hr (yes/no) at study enrollment. A post-shift questionnaire was administered to a subset of cashiers to assess hours worked at a register, average number of transactions, consumption of food or beverage from metal containers, use of polycarbonate plastic, frequency of hand washing, and use of gloves and hand creams during work. All human subject research activities were conducted at the National Institute of Environmental Health Sciences (NIEHS) Clinical Research Unit (CRU) in accordance with protocols approved by the NIEHS Institutional Review Board (IRB #10-E-0063), and all participants gave written informed consent before providing their medical history and donating samples. Participation of the National Center for Toxicological Research (NCTR) laboratory was reviewed and approved by the U.S. Food and Drug Administration (FDA) Research Involving Human Subjects Committee (RIHSC #11-067T).

*Receipt, blood, and urine sample collection.* Care was taken to avoid possible BPA contamination from laboratory materials and equipment by using glass pipets and polypropylene containers, including water blanks for blood and urine collection and processing procedures, and providing special instructions to CRU staff.

Each cashier provided a receipt sample from her/his place of employment that was at least 12 in long and was stored in a Ziploc® bag (these bags do not contain BPA). Two sets of samples were collected from each cashier, one “post-shift” sample collected within 2 hr of completing a work shift, and one “pre-shift” sample collected at least 24 hr after a work shift had been completed. To accommodate cashier work schedules, sample collections did not have to occur before and after the same work shift, and in ~ 30% of cashiers, the post-shift sample was the first sample collected (see Supplemental Material, Table S1, for complete study data from each participant). We initially intended to have both visits occur on the same day but found this to present a significant challenge to participant recruitment. Thus, to accommodate cashier work schedules, the “pre-shift” sample was collected at the CRU at a visit that occurred after being off-duty for at least 24 hr. The study participants were not required to fast or to avoid specific food items or consumer products. A single urine sample was collected from each non-cashier at the CRU during normal CRU business hours (0800–1630 hours).

Blood samples were taken by trained phlebotomists using a 1-in (2.54 cm), 22-gauge metal needle (Becton Dickinson) attached to a disposable polypropylene tube holder (Becton Dickinson Vacutainer). Blood was collected into a 10-mL nonsiliconized “red-top” glass blood collection tube without clot activators or other additives (Becton Dickinson). Samples were allowed to clot at room temperature for at least 60 min, centrifuged at 300 × *g* for 10 min, and serum was transferred into 1.5-mL polypropylene microcentrifuge tubes (Sarstedt) using disposable glass pipets (Kimble Chase). Samples were stored at –80°C, shipped on dry ice to NCTR for analysis, and stored at –60°C until analyzed.

Urine samples were collected in polypropylene collection cups (Andwin Scientific). Water blanks using high-performance liquid chromatography (HPLC)–grade water were prepared in the same manner and collection containers as the blood and urine samples. One-milliliter samples of serum, urine, and two water blanks (one for blood, one for urine) were aliquoted into four 1.5-mL polypropylene microcentrifuge tubes (Sarstedt) for storage. Samples were stored at –80°C, shipped on dry ice to NCTR for analysis, and stored at –60°C until analyzed.

*Materials used for analytical chemistry.* All HPLC solvents including water were Optima liquid chromatography/mass spectrometry (LC/MS) grade and were purchased from Fisher Scientific, except for methanol, which was purchased from JT Baker. Native BPA, β-glucuronidase/arylsulfatase (*Helix pomatia*, H1, 16 units/mg), ^13^C_12_-BPS (> 99% isotopic purity), and all other chemical reagents were purchased from Sigma Aldrich. ^13^C_12_-BPA (> 99% isotopic purity) was obtained from Cambridge Isotope Labs, unlabeled BPA-glucuronide (BPA-G), and ^13^C_12_-BPA-G (> 99% isotopic purity) were prepared and provided by the National Toxicology Program (NTP), ^13^C_6_-BPS was purchased from Toronto Research Chemicals, and BPSIP (98% purity) was purchased from AK Scientific. Control Sprague-Dawley rat serum (not filtered) was purchased from Bioreclamation LLC (Westbury, NY), and the control urine sample was provided by a human volunteer in our laboratory.

*Receipt analysis.* Receipts were analyzed for extractable compounds as follows: A 100-mg portion of a receipt was placed into 10 mL methanol, which was then placed into an ultrasonic bath for 30 min. The methanol-extractable components were evaluated using liquid chromatography–ultraviolet detection (LC-UV) (280 nm) and full-scan LC/MS (positive and negative ion detection). The only methanol-extractable compounds detected were BPA, BPS, and BPSIP, and they were identified by comparison of retention time and full-scan mass spectral data with authentic standards for BPA and BPS (not shown). The amount of each compound present in each receipt was then quantified using liquid chromatography–tandem mass spectrometry (LC/MS/MS) with internal standard calibration for BPA and BPS and external standard calibration for BPSIP (^13^C_12_-BPA). The levels of detection were 0.2 mg BPA/g paper, 0.02 mg BPS/g paper, and 0.07 mg BPSIP/g paper.

*Sample preparation.* Serum. Serum samples for measurement of unconjugated and total BPA were prepared as previously described using liquid-liquid extraction (Churchwell et al. 2014; Teeguarden et al. 2011). Each serum sample was processed identically to measure both unconjugated and total BPS and BPSIP.

For cashier serum samples found to contain total BPA, BPS, or BPSIP, the unconjugated form was also analyzed to evaluate possible post-sampling contamination. Further evaluation of possible BPA contamination was conducted by directly quantifying the individual conjugates BPA-G and BPA-S (Churchwell et al. 2014; Teeguarden et al. 2011). Serum samples from non-cashiers were not analyzed.

Urine. Urine samples to be used for measurement of total and unconjugated BPA were prepared as previously described (Churchwell et al. 2014; Teeguarden et al. 2011). Urine samples to be used for total BPS and BPSIP measurements were prepared similarly to the serum samples except that acetonitrile was used in place of methyl *tert*-butyl ether (MTBE). Urine samples to be used for measurements of unconjugated BPS and BPSIP were prepared as follows: 100 μL of urine, 100 μL of water, and 50 μL of internal standard were mixed in a deactivated Max Recovery vial. The vial was briefly mixed and then shaken on a 23°C thermomixer for 10 min at 1,400 RPM. The vial was centrifuged at 10,000 RPM for 10 min and stored in a –20°C freezer for 30 min. The acetonitrile layer was transferred to a new deactivated vial with a Pasteur pipet and evaporated to dryness at reduced pressure using a heated centrifugal concentrator. The urine samples were reconstituted in the same manner as the serum samples. For totals analysis, 100 μL of urine, 100 μL of β-glucuronidase/arylsulfatase (1 mg/mL in 25 mM citrate buffer, pH 5), and 50 μL of internal standard were gently mixed in a deactivated vial and then incubated at 37°C for 2 hr. The remaining preparation steps were the same as for unconjugated analysis. Urine creatinine levels were determined at the Department of Laboratory Medicine, NIH Clinical Center, as a Clinical Laboratory Improvement Amendments (CLIA)-certified test using a Siemens Dimension EXL analyzer. Urine was stored at –80°C until testing.

*Characterization and preparation of standards.* Characterization of the ^13^C_12_-BPA was performed as described by Teeguarden et al. (2011). LC-UV (Dionex AD20, 280 nm) was used to verify the concentration of unlabeled and labeled BPS standards. A Luna analytical column (2.0 × 150 mm, 3 μm particle, Phenomenex) was used at a flow rate of 0.2 mL/min with an isocratic mobile phase consisting of 40% aqueous acetonitrile. Isotopic purity for the labeled BPS was 90%, and no unlabeled BPS was detected by LC/MS/MS (< 0.1%). The BPSIP was prepared from solid material and used as weighed.

Working standard and internal standard solutions for BPS and BPSIP were prepared in 50% acetonitrile/50% water. Pools of control rat serum and spiked control rat serum or urine were prepared for use as daily quality-control samples. In addition to the quality-control samples, four enzyme blanks or four unconjugated blanks were prepared along with each sample set to establish background BPS and BPSIP levels.

*LC/MS/MS determinations in urine and serum.* BPA. LC/MS/MS was used with on-line column switching to analyze total and unconjugated native BPA in urine and serum as reported previously (Churchwell et al. 2014; Teeguarden et al. 2011). Limits of detection (LODs) were determined daily for BPA in urine (0.07–0.25 ng/mL) and for BPA in serum (0.045– 0.35 ng/mL). BPA conjugates were analyzed in serum to confirm positive findings of total BPA, and the LODs for BPA-G and BPA-S were 0.04 and 0.06 ng/mL, respectively, for 100-μL aliquots.

BPS. The liquid handling system consisted of an Acquity UPLC system (Waters Inc.), a 1260 Infinity HPLC pump (Agilent), and an automated six-port switching valve (Rheodyne). The system also had a Luna C18(2) column (2.0 × 30 mm, 3 μm particle size, Phenomenex) installed between the binary solvent manager and the sample manager. The on-line SPE column was a Shodex ODP2 HP (2 × 50 mm, macroporous particle type; ES Industries), and the HPLC column was a Shodex ODP2 HP (2 × 150 mm). The analytical column was maintained at 45°C. The Acquity system was used to load 50 μL of sample on the SPE column and to wash the SPE column. The Agilent pump eluted the sample components from the SPE column to the analytical column and maintained a constant flow of mobile phase into the mass spectrometer during sample-loading periods. The switching valve was used to divert the column effluent to either waste or the analytical column. The sample was loaded at 0.3 mL/min for 5.0 min with 80% water/20% methanol. After switching the divert valve, the concentrated sample zone was back-flushed to the analytical column with 60% water/40% acetonitrile at 0.2 mL/min for 2 min. At 2.1 min, a linear gradient raised the acetonitrile concentration to 90% over 10 min and then held steady from 12 to 14 min. At 14 min, the gradient was reset to initial conditions. The SPE column was in-line with the analytical column from 5.1 to 6.2 min. From 8.9 to 14.9 min, the SPE column was cleaned with 95% methanol/5% water. At 15 min, the Acquity gradient was reset to the initial conditions. The total run time including sample loading was 22 min.

A Xevo TQ-S triple quadrupole mass spectrometer (Waters) equipped with an electrospray ionization (ESI) source was used in selected reaction monitoring mode for analysis of negative ions. Capillary voltage was 2.5 kV, and the cone gas flow rate was 150 L/hr. Other MS parameters included source and desolvation temperatures of 150°C and 500°C, respectively, argon as the collision gas (0.17 mL/min), and nitrogen as the desolvation gas (1,000 L/hr). Two transitions were monitored for both the labeled and unlabeled BPS. A cone voltage of 45 V was used for all transitions. LODs were determined daily: BPS urine (0.01–0.02 ng/mL) and BPS serum (0.002–0.01 ng/mL).

BPSIP. BPSIP was analyzed in urine and serum using the LC conditions described above for BPS. Concentrations of BPSIP were initially evaluated using ^13^C_12_-BPA as a surrogate internal standard. The method performance was evaluated during the BPS validation procedure using control and spiked matrices. The validation procedure produced acceptable precision and accuracy ranges. However, when actual cashier serum or urine was analyzed, the method failed because of the wide range of suppression observed on the^13^C_12_-BPA, which did not affect the BPSIP. Because no other suitable internal standard could be identified for quantification of BPSIP, semi-quantitative results were evaluated as either above or below the LOD for urine (0.01–0.02 ng/mL) and serum (0.005–0.008 ng/mL). Subsequently, all urine samples containing total BPSIP above the daily limit of quantitation (LOQ) (0.03–0.06 ng/mL) were quantified using the method of standard addition, where two aliquots of each sample were analyzed: One aliquot was spiked with a known amount of BPSIP matched to the target concentration, which was based on the value estimated from the original analysis; the other aliquot was not spiked. The control human urine sample from the laboratory volunteer was also analyzed in duplicate with standard addition to provide a background value of contamination during sample preparation. The amount of BPSIP was quantified by dividing the area under the chromatographic peak of the unspiked sample with the area under the chromatographic peak of the spiked sample minus the area of the unspiked sample and multiplying by the amount of BPSIP added in ng/mL. The background value generated from the control urine was subtracted from each sample before results were reported. All serum samples contained total BPSIP below the LOQ (0.015–0.024 ng/mL) and were not analyzed further.

*Method validation and quality control.* BPA. The validation of the on-line column switching LC/MS/MS method was reported previously (Teeguarden et al. 2011). Measurable responses for BPA were observed in all procedural blanks because trace level contamination by native BPA is difficult to avoid (Teeguarden et al. 2011; Twaddle et al. 2010; Ye et al. 2013). Accordingly, four replicate procedural blanks were analyzed with each sample set to determine a daily limit of blank (LOB). These samples, which consisted of water instead of serum, were subjected to the entire sample preparation process. The LOB was defined as the mean value of the replicates plus two standard deviations, and the daily LOB was subtracted from each serum sample concentration (with enzymatic hydrolysis, 0.5–1.8 nM; without enzyme, 0.3–1.1 nM). In addition, daily LODs were estimated from the amount of BPA producing a signal/noise ratio > 3 above the LOB (with enzymatic hydrolysis, 0.2–1.1 nM, without enzyme, 0.1–0.4 nM). If the sample quantification value after subtraction of the LOB was not higher than the daily calculated LOD, it was reported as < LOD. Intra- and inter-day precision ranged from 0.6–5.3% relative standard deviation (RSD). Intra- and inter-day accuracy ranged from 98% to 105%. Accuracy was defined as the percentage of how close the calculated value for a spiked control sample came to the actual known spiked amount.

BPS and BPSIP. Calibration curves were generated for BPS by adding varying concentrations of unlabeled BPS while keeping the internal standard concentration constant. The curve was linear over the range of 0–10 ng/mL with a slope of 0.89. The serum and urine methods were validated over 2 days using control serum, spiked control serum, and incurred study serum. Sprague Dawley rat serum was also used as control serum for the BPS and BPSIP methods. An incurred BPS study serum was prepared by adding a small amount of a previously analyzed BPS urine sample with a known total BPS level to a large volume of control serum. This sample was also spiked with a known amount of BPSIP. The use of the incurred study serum validated that the enzyme worked properly for analysis of total BPS. Validation was performed on 100-μL aliquots of serum and urine. Intra- and inter-day precision (RSD) ranged from 0.8% to 12.2%. Intra- and inter-day accuracy ranged from 93% to 107%. Control serum was spiked at 0.1 ng/mL for analyses of unconjugated and total compounds in 100-μL serum samples. Control urine was spiked at 0.1 ng/mL and 1.0 ng/mL for the validation of levels of both total and unconjugated compounds.

Duplicates of control serum and pooled incurred serum were analyzed with each serum sample set as quality control checks. The incurred study serum prepared for the validation was also used as an incurred serum for daily BPS and BPSIP analyses. Duplicates of control and spiked urine were analyzed with each urine sample set. In addition, four replicate method blanks were analyzed with each sample set. In these blanks, water was used in place of serum, and they were subjected to the entire sample preparation process. These samples provided a measurement of background BPS generated during sample preparation (i.e., LOB). The average concentration value of the replicates plus two standard deviations was subtracted from each sample concentration, and the difference was reported as the sample concentration. In addition, daily LODs were generated from calculating the signal-to-noise ratio of several different serum or urine samples with low calculated BPS or BPSIP values. Because of the wide variation of ion suppression that was observed between individual serum or urine samples, a daily LOD for a signal-to-noise ratio of 3–4 was generated based on an average of these observations. If the sample value after subtraction of the background was not higher than the daily calculated LOD, it was reported as < LOD.

*Assessment of potential BPA contamination.* Samples were considered to show evidence of possible BPA contamination when high percentages of BPA were present in unconjugated form (≥ 20%) based on analysis with and without complete enzymatic hydrolysis. Direct analysis of individual BPA conjugates, BPA-G and BPA-S, was also performed because conjugates are the predominant species present in serum and urine after either oral (> 99% of total BPA) or parenteral administration (> 85% of total) (NTP 2008, Doerge et al. 2010). Samples in which ≥ 20% of total BPA was present as unconjugated and no BPA-G (LOD = 0.04ng/mL) or BPA-S (LOD = 0.06 ng/mL) conjugate was detected were classified as suspected contamination (see Supplemental Material, Table S1).

*Statistical analysis.* Results of the receipt paper analysis expressed as percentage of total paper weight showed that the receipts contained 1–2% of BPA, BPS, or BPSIP. We assigned cashiers to receipt groups based on the dominant analyte detected in the receipt paper. Post-shift urine levels of total BPA, BPS, and BPSIP in cashiers were compared with pre-shift levels and with levels in samples collected from 25 non-cashiers. Analysis of urine BPA and BPS was quantitative, whereas BPSIP analysis was frequency based; that is, the results were reported as either above or below the LOD because most urine samples did not have BPSIP levels > LOQ. We also conducted frequency-based analyses (< LOD vs. > LOD) of serum levels of total BPA and BPS in pre- and post-shift samples from cashiers and of BPSIP in a subset of cashier samples. Serum BPA and BPS were not measured in non-cashiers because the frequency of detection was low in cashiers.

Statistical analysis was conducted using SAS version 9.3 (SAS Institute Inc., Cary, NC). Creatinine-adjusted urine levels were natural log–transformed during statistical analysis because they were right-skewed. When the level was < LOD, a value of LOD/2 was used for BPA and BPS quantitation; use of this value is considered reasonable when the proportion of samples below the LOD is relatively small (< 15%), as was the case in the present study (Gillespie et al. 2005). We did not impute values < LOQ for urine BPSIP because the levels in many samples were below the LOD or LOQ. Paired *t*-tests were used to compare pre- and post-shift urine levels of BPA or BPS. Two-sample *t*-tests were used to compare mean urine concentrations in non-cashiers with mean urine concentrations in BPA- or BPS-exposed cashiers, respectively. Frequency of detection data were compared for pre- and post-shift levels using McNemar’s chi-squared test and were compared between groups of participants using chi-squared or Fisher’s exact tests. One-sided *p*-values were used because we hypothesized *a priori* that post-shift, cashiers would have higher levels/detection frequency of the developer used in the receipts they handled than they would have pre-shift, and that the levels/detection frequency in cashiers would be higher than in non-cashiers. *p*-Values < 0.05 were considered statistically significant. We used stepwise regression with an entry significance level of 0.15 and an exit significance level of 0.10 to determine whether fasting status, defined as eating or drinking in the 8 hr preceding sample collection, and shift sequence (i.e., whether the post-shift sample was collected first) were predictors of changes in BPA and BPS concentrations in urine between pre-shift and post-shift collections. Fasting status and shift sequence data were available for most subjects and were included in all models and not subject to removal from any model.

## Results

Selected data from individual study participants included in this analysis are provided in Supplemental Material, Table S1.

*Participants.* A total of 91 male and female cashiers 19–77 years old were recruited from restaurants, grocery stores, pharmacies, clothing stores, bookstores, and home improvement centers. Six were excluded because they did not complete both visits, and 7 were excluded because they did not provide a receipt sample, the receipt paper was of poor physical quality and not analyzable, or the sample was not thermal paper. One additional cashier was excluded because the pre-shift urine creatinine result was unusually low (0 g creatinine). Thus, a total of 77 cashiers were included in the analysis. Cashiers were grouped into receipt categories based on the dominant analyte detected in the paper (BPA = 33, BPS = 32, BPSIP = 12) (Tables 1 and 2). Urine samples were also collected from 25 non-cashiers.

**Table 1:** Receipt characteristics.

Receipt category	*n*	BPA content (mg/g paper)^*a*^	BPS content (mg/g paper)^*a*^	BPSIP content (mg/g paper)^*a*^
BPA	33	19.6 ± 4.7 (mean ± SD) 19.3 (median) 7.0–36.0 (range)	2/34 (6%) > LOD maximum = 1.09	0/33 (0%) > LOD
BPS	32	1/32 (3%) > LOD maximum = 0.81	15.0 ± 2.6 (mean ± SD) 14.6 (median) 11.9–26.2 (range)	0/32 (0%) > LOD
BPSIP	12	1/12 (8%) > LOD maximum = 0.70	6/12 (50%) > LOD maximum = 0.05	13.5 ± 0.9 (mean ± SD) 13.9 (median) 12.4–14.8 (range)
Non-cashiers	25	NA	NA	NA
Abbreviations: BPA, bisphenol A; BPS, bisphenol S; BPSIP, 4-hydroxyphenyl 4-isoprooxyphenylsulfone; LOD, limit of detection; NA, not applicable. LODs were 0.2 mg BPA/g paper, 0.02 mg BPS/g paper, and 0.07 mg BPSIP/g paper. ^***a***^Divide by 10 to convert mg/g paper to percent of paper weight.				

**Table 2:** Demographic characteristics of study participants.

Receipt category	*n*	Sex (% male)	Age (years) mean ± SD median (range)	BMI (kg/m^2^) mean ± SD median (range)	Race
BPA	33	20.6	35.0 ± 12.7 30.1 (19.8–65.0)	29.0 ± 5.8 27.8 (20.1–43.0)	48% black; 39% white; 3% Asian; 9% multiple
BPS	32	41.9	35.9 ± 14.4 33.2 (19.8–77.5)	29.9 ± 8.0 27.1 (18.0–46.0)	38% black; 50% white; 3% Asian; 6% multiple; 3% unknown
BPSIP	12	50.0	40.4 ± 13.6 40.7 (22.5–60.6)	26.5 ± 5.4 25.1 (19.0–35.1)	25% black; 58% white; 17% multiple
Non-cashiers	25	60.0	44.9 ± 12.4 51.3 (23.1–63.9)	27.9 ± 5.0 28.0 (19.8–38.6)	24% black; 60% white; 16% unknown
Abbreviations: BMI, body mass index; BPA, bisphenol A; BPS, bisphenol S; BPSIP, 4-hydroxyphenyl 4-isoprooxyphenylsulfone. Additional medical history information such as menopausal status, smoking, alcohol use, medications, and disease status available in Supplemental Material, Table S1.					

Information is available regarding CRU visit dates and fasting status before sample collection for all of the cashiers and for 24 of 25 non-cashiers (see Supplemental Material, Table S1). The interval between collection of pre-shift and post-shift samples ranged from the same day to several months and was less than 1 week for almost 70% of cashiers. Most of the cashiers (62/77 pre-shift; 69/77 post-shift) and non-cashiers (20/25) did not fast in the 8 hr before sample collection. An insufficient number of cashier participants (27/77) completed a separate post-shift questionnaire to support quantitative analysis of factors such as length of shift, average number of transactions during the shift, consumption of metal-canned foods or drinks, use of polycarbonate food packaging, use of gloves and hand creams, and degree of hand washing.

*Receipt samples.* Only one analyte was the dominant form in the thermal receipt paper samples; levels of the other analytes were either non-detectable or only detected in amounts < 0.1% by weight in the paper tested (Table 1).

**Figure 2:**
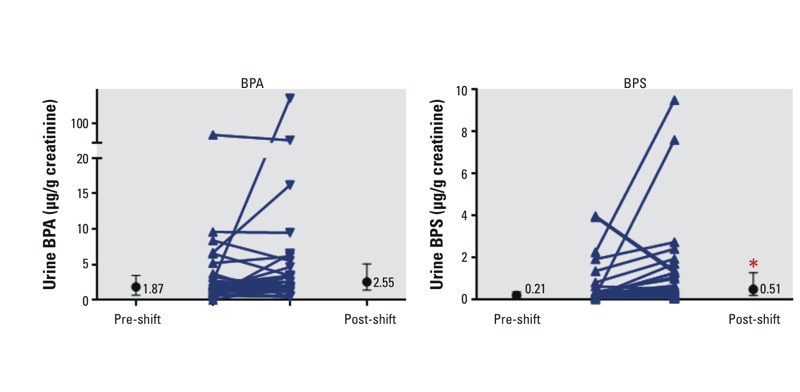
Pre- and post-shift urinary levels of BPA and BPS: individual patterns and group median. Error bars for the group medians indicate the 25–75% range. *p < 0.001, significant difference between pre-shift and post-shift.

*Urine levels of total BPA, BPS, and BPSIP.* Although post-shift levels of urinary BPA tended to be higher than pre-shift levels in cashiers who handled BPA receipt paper [geometric mean (SD): pre-shift = 1.89 (3.63) μg/g; post-shift = 2.76 (3.53) μg/g; Table 3], the difference was not statistically significant (*p* = 0.10). There was considerable variability within individual cashiers; post-shift urine levels of BPA were actually lower than pre-shift levels in almost half of cashiers handling BPA-containing receipts (Figure 2; see also Supplemental Material, Table S1). Post-shift urine levels of BPA in the BPA–receipt paper cashier group were significantly higher than levels in non-cashiers [geometric mean (SD): 1.25 (1.79) μg/g; post-shift *p* < 0.001]. Urine levels of BPA in the samples from non-cashiers were slightly lower than in the most recent NHANES 2011–2012 data (geometric mean of 1.72 μg/g creatinine) [Centers for Disease Control and Prevention (CDC) 2015]. In the step-wise regression analysis, neither shift sequence nor fasting status was a significant predictor variable for differences in pre-shift versus post-shift levels of BPA (data not shown).

**Table 3:** Urine total BPA, BPS, and BPSIP in cashiers and non-cashiers.

Compound	Geometric mean (SD) [range] (μg/g creatinine)			
	Cashiers, BPA receipts *n *= 33	Cashiers, BPS receipts *n *= 31	Cashiers, BPSIP receipts *n *= 12	Non-cashiers *n *= 21
BPA urine
Cashiers, pre-shift	1.89 (3.63) [< LOD–57.56]	1.33 (2.89) [0.19–41.22]	0.71 (2.85) [< LOD–2.80]	NA
Cashiers, post-shift	2.76 (3.53)** [0.44–187.96]	1.35 (2.34) [0.29–20.38]	1.07 (2.01) [0.37–4.41]	NA
Non-cashiers	NA	NA	NA	1.25 (1.79) [< LOD–4.19]^*a*^
BPS urine
Cashiers, pre-shift	0.31 (3.64) [< LOD–4.36]	0.23 (3.89) [< LOD–3.99]	0.38 (3.75) [< LOD–2.16]	NA
Cashiers, post-shift	0.25 (3.16) [0.13–3.48]	0.54 (3.62)* [0.53–9.50]	0.28 (3.06) [< LOD–3.47]	NA
Non-cashiers	NA	NA	NA	0.41 (5.26) [< LOD–11.04]
BPSIP urine
Cashiers, pre-shift	4/33 (12.1%) [all < LOQ]	6/32 (18.8%) [all < LOQ]	10/12 (83.3%) ** [< LOD–0.272]	NA
Cashiers, post-shift	6/33 (18.2%) [< LOD–0.035]	9/32 (28.1%) [< LOD–0.762]	9/12 (75.0%) ** [< LOD–1.19]	NA
Non-cashiers	NA	NA	NA	8/25 (32.0%) [< LOD–0.139]
Abbreviations: BPA, bisphenol A; BPS, bisphenol S; BPSIP, 4-hydroxyphenyl 4-isoprooxyphenylsulfone; LOD, limit of detection; LOQ, limit of quantitation; NA, not applicable. Urine LODs: BPA, 0.07–0.25 ng/mL; BPS, 0.01–0.02 ng/mL; BPSIP, 0.01–0.02 ng/mL. ^***a***^For comparison, the geometric mean level of BPA from NHANES 2011–2012 is 1.72 g/g creatinine (CDC 2015). *p < 0.001, significant difference between pre-shift and post-shift; **p < 0.02, significant difference compared with non-cashiers.				

Post-shift levels of total urinary BPS were significantly higher than pre-shift levels for the 32 cashiers who handled BPS-containing receipts [geometric mean (SD) pre-shift = 0.23 (3.89) μg/g, post-shift = 0.54 (3.62) μg/g; *p* < 0.001; Table 3; see also Supplemental Material, Table S1]. Levels of BPS were higher in post-shift samples than in pre-shift samples for most of the cashiers in the BPS–receipt paper group (26/32, Figure 2). Neither pre-shift nor post-shift urine levels of BPS in these cashiers were significantly higher than levels in non-cashiers (geometric mean (SD): 0.41 (5.26) μg/g). Neither shift sequence nor fasting status was a significant predictor variable for differences in pre-shift versus post-shift levels of BPS in the step-wise regression analysis (data not shown).

In the 12 cashiers who handled BPSIP-containing receipts, the proportion of samples with detectable BPSIP was similar in pre- and post-shift samples (10/12, 83% and 9/12, 75%, respectively; *p* = 0.65) (Table 3). BPSIP was detected more frequently in cashiers in the BPSIP group pre- and post-shift than in cashiers in the other receipt groups, where the pre- and post-shift detection frequency ranged from 12.1 to 28.2% (*p* < 0.02; Table 3). BPSIP was also detected significantly less often in non-cashiers [32% (8/25); *p* < 0.02]. BPSIP concentrations were > LOQ in 58% and 67% of pre- and post-shift samples from the BPSIP cashier group, respectively, compared with 0–16% of samples from other cashier groups and from non-cashiers.

*Serum levels of total BPA, BPS, and BPSIP.* In the BPA-receipt group, most cashiers had pre- and post-shift levels of total serum BPA that were < LOD or < LOQ (26/33, or 79%, in both pre- and post-shift samples) (Table 4). Contamination was suspected in 5 of the 6 serum samples with BPA > LOQ in the BPA-receipt group based on the sample having a relatively high fraction of total BPA present in unconjugated form (> 20%) (see Supplemental Material, Table S1). The presence of BPA-G and BPA-S was confirmed in the samples containing measurable total BPA but was not observed in samples showing high percentages of unconjugated BPA (data not shown), which is also indicative of contamination. BPA was also typically < LOD or < LOQ in cashiers in the BPS–receipt paper group (88% at both time points) and the BPSIP–receipt paper group (66% pre-shift; 100% post-shift) (Table 4).

**Table 4:** Serum total BPA, BPS, and BPSIP in cashiers.

Compound	BPA receipts (number > LOD)	BPS receipts (number > LOD)	BPSIP receipts (number > LOD)
BPA serum
Pre-shift	7/33 (21.2%)	4/32 (12.5%)	4/12 (33.3%)
Post-shift	7/33 (21.2%)	4/32 (12.5%)	0/12 (0%)
BPS serum
Pre-shift	9/33 (27.3%)	5/32 (15.6%)	2/12 (16.7%)
Post-shift	5/33 (15.2%)	13/32 (40.6%)*	1/12 (8.3%)
BPSIP serum
Pre-shift	9/21 (42.9%)	5/15 (33.3%)	7/12 (58.3%)
Post-shift	6/17 (35.3%)	7/16 (43.8%)	6/12 (50.0%)
Abbreviations: BPA, bisphenol A; BPS, bisphenol S; BPSIP, 4-hydroxyphenyl 4-isoprooxyphenylsulfone; LOD, limit of detection. Serum LODs: BPA, 0.045–0.35 ng/mL; BPS, (0.002–0.01 ng/mL; BPSIP, 0.005–0.008 ng/mL. *p = 0.02, significant difference between pre-shift and post-shift.			

In the BPS–receipt paper group, serum total BPS was detected significantly more frequently in post-shift samples than in pre-shift samples (13/32 or 40.6% post-shift vs. 5/32 or 15.6% pre-shift, *p* = 0.02). Most of the 18 samples that had detectable levels in the BPS–receipt paper group (i.e., > LOD) were < LOQ. Detectable levels were also measured in serum samples from cashiers in the BPA–receipt paper (14/66) and the BPSIP–receipt paper (3/24) groups.

Serum BPSIP was detected in cashiers from the BPSIP–receipt paper group at levels between the LOD and LOQ, but the detection frequency did not differ between pre- and post-shift samples (7/12 or 58.3% post-shift vs. 6/12 or 50% pre-shift). BPSIP was also detected in 33–44% of samples from cashiers in the BPA– and BPS–receipt paper groups (Table 4). Furthermore, BPSIP was more consistently detected in a greater percentage of samples in cashier groups (33–58.3%) than was BPA (0–33%) or BPS (8.3–40.6%).

## Discussion

In aggregate, our results support occupational use of thermal paper as a source of exposure to BPA, BPS, and BPSIP. However, there was considerable within-subject variability, especially for BPA; that is, levels were often lower in post-shift samples than in pre-shift samples (Figure 2). We did not have a sufficient number of completed post-shift questionnaires to support statistical analyses on which factors might predict patterns of response (length of shift, average number of transactions during the shift, consumption of canned foods or drinks, use of polycarbonate food packaging, use of gloves and hand creams, and degree of hand washing). Based on the questionnaire data we have (completed by ~30–40% per group), most cashiers did not use gloves, did wash their hands regularly during shifts, reported infrequent use of hand creams (one or fewer times during shifts), and did not eat or drink often from metal food cans or polycarbonate plastic food containers. Only one cashier in the BPA-receipt group reported eating or drinking multiple times from a metal food can or a plastic food container during a shift. Most cashiers reported engaging in one transaction every 5 to 10 min, but some reported > 1 per minute, and others reported ≤1 per 30 min.

Our analysis of receipt content of BPA and BPS found levels of these compounds similar to those reported in other studies (Biedermann et al. 2010; Geens et al. 2012; Lassen et al. 2011; Liao and Kannan 2011; Liao et al. 2012c; Lu et al. 2013; Lunder et al. 2010; Mendum et al. 2011; Östberg and Noaksson 2010; Schreder 2010). We observed one predominant compound (BPA, BPS, or BPSIP) in each sample of thermal paper receipts, suggesting that only one of the compounds was used as the primary developer for any receipt. BPA and BPS are known to be used in thermal paper (Liao et al. 2012c; U.S. EPA 2014), but this is the first confirmed use of BPSIP, which was found in receipts collected from 12 cashiers working at two retailers.

Exposure via contact with thermal paper could occur through dermal or non-dermal routes. Dermal uptake is possible, but other potential pathways of exposure for cashiers include ingestion and inhalation of dust particles containing the compounds and inhalation if the compounds become volatile. Studies designed to model cashier exposure suggest that extensive dermal contact is needed in order to produce a detectable post-handling increase in BPA (e.g., receipts handled continuously without gloves for 2 hr) (Ehrlich et al. 2014) or firmly rubbing the paper for several minutes repeatedly (Porras et al. 2014). Patterns of extensive handling of receipts are unlikely to occur routinely in cashiers, and contact is more likely to be intermittent and to last only seconds at a time (Lassen et al. 2011), highlighting the importance of considering nondermal exposure pathways. The current study focused on cashiers, but other occupations involving potentially high exposures should also be considered. For example, BPA can be found in medical apparatus thermal paper at levels similar to those in cash register receipts (Östberg and Noaksson 2010).

We measured detectable levels of BPS and BPSIP in urine from non-cashiers. The BPS result is not surprising given that BPS has been reported in urine in the general population (Liao et al. 2012a) and can also be found in food (Liao and Kannan 2013), personal care products (Liao and Kannan 2014), dust (Liao et al. 2012b), soil sediment (Liao et al. 2012d), and paper products such as currency, tickets, and airplane boarding passes (Liao et al. 2012c). Very little is known about uses of BPSIP outside of its use as an alternative to BPA in thermal paper (U.S. EPA 2014). To the best of our knowledge, no other study has reported information on its detection in human or environmental samples, food, or receipts. BPSIP was detected in the serum samples from cashiers more often than BPA or BPS, regardless of whether BPSIP was the predominant compound in the receipts they handled (Table 4), which raises questions about whether it may be more environmentally persistent, less readily cleared from the body, and whether exposure is more widespread than previously assumed. We did not measure BPSIP or the other compounds in serum samples from non-cashiers.

Ten serum samples had quantifiable levels (> LOQ) of total BPA (see Supplemental Material, Table S1). However, contamination was suspected in 7 of these samples based on the presence of a relatively high portion of the total BPA in unconjugated form (≥ 20%) and the absence of detectable BPA conjugates (BPA-G and BPA-S). Sample contamination by BPA has been widely reported, even when steps are taken to minimize potential contamination during sample collection and analysis, as was done in the present study (Calafat et al. 2013; Longnecker et al. 2013; Teeguarden et al. 2013; Twaddle et al. 2010; Ye et al. 2013). In contrast, there were few indications of sample contamination in our serum BPS and BPSIP analyses, perhaps reflecting their more limited usage in laboratory materials used for sample collection, sample storage, and analytical chemistry.

There are limitations to this study. Sample sizes were small in each group, and our study was not designed to discern which routes of exposure might account for the observed patterns in cashiers, that is, dermal, oral, and/or inhalational, or to rule out exposure from other sources. Furthermore, there is uncertainty about the pharmacokinetics of these compounds following dermal exposure, and collection of samples ≤ 2 hr after the particpants’ shifts may not have been ideal for detecting peak levels. A portion of BPA may be retained in the skin following dermal contact (Demierre et al. 2012; Kaddar et al. 2008; Mørck et al. 2010), and it may take more than a day for this portion to be taken up through the skin into systemic circulation and eliminated via urine (Marquet et al. 2011). In another study published after ours was initiated (Ehrlich et al. 2014), the highest levels of BPA in urine occurred 6–10 hr after handling of receipts, at levels approximately twice as high as when urine was collected 2 hr post-handling. Another potential limitation is that the pre-shift visit did not necessarily occur on the same day as the post-shift visit (although most occurred during the same week), and the post-shift visit occurred prior to the pre-shift visit in ~ 30% of participants. In addition, we did not know the time interval between last receipt handling and sample collection in the post-shift samples, and it is possible that 24 hr of not handling receipts before pre-shift sample collection may not be a sufficient washout period for BPA levels to return to baseline. Furthermore, we did not know how many hours were worked during the workweek preceding the post-shift visit. These factors may explain why many cashiers in the BPA–receipt paper group had lower post-shift than pre-shift levels; that is, sources of exposure other than occupational sources might have had a greater influence on urine BPA levels. We also did not attempt to limit exposures to BPA from other sources, such as food or drink. Additional studies would be needed to address these limitations.

## Conclusion

In conclusion, our results indicate that thermal paper is a potential source of exposure to BPA and similar compounds for cashiers and may be a source of exposure for other occupations in which frequent contact with thermal paper occurs.

## Supplemental Material

(438 KB) ZIP
